# Catalytic reactor for *operando* spatially resolved structure–activity profiling using high-energy X-ray diffraction

**DOI:** 10.1107/S1600577523001613

**Published:** 2023-04-12

**Authors:** Birte Wollak, Diego Espinoza, Ann-Christin Dippel, Marina Sturm, Filip Vrljic, Olof Gutowski, Ida G. Nielsen, Thomas L. Sheppard, Oliver Korup, Raimund Horn

**Affiliations:** a Institute of Chemical Reaction Engineering (CRT), Hamburg University of Technology (TUHH), Eißendorfer Straße 38, 21073 Hamburg, Germany; b Deutsches Elektronen-Synchrotron DESY, Notkestraße 85, 22607 Hamburg, Germany; c Institute of Catalysis Research and Technology (IKFT), Karlsruhe Institute of Technology (KIT), Hermann-von-Helmholtz-Platz 1, Eggenstein-Leopoldshafen, 76344 Baden-Württemberg, Germany; d REACNOSTICS GmbH, Am Kaiserkai 30, 20457 Hamburg, Germany; Bhabha Atomic Research Centre, India

**Keywords:** heterogeneous catalysis, *operando* profile measurements, structure–activity relationships, catalytic reactors

## Abstract

In heterogeneous catalysis, *operando* measurements probe catalysts in their active state and are essential for revealing complex catalyst structure–activity relationships. The development of appropriate *operando* sample environments for spatially resolved studies has come strongly into focus in recent years, particularly when coupled to the powerful and multimodal characterization tools available at synchrotron light sources. However, most catalysis studies at synchrotron facilities only measure structural information about the catalyst in a spatially resolved manner, whereas gas analysis is restricted to the reactor outlet. Here, a fully automated and integrated catalytic profile reactor setup is shown for the combined measurement of temperature, gas composition and high-energy X-ray diffraction (XRD) profiles, using the oxidative de­hydrogenation of C_2_H_6_ to C_2_H_4_ over MoO_3_/γ-Al_2_O_3_ as a test system. The profile reactor methodology was previously developed for X-ray absorption spectroscopy and is here extended for *operando* XRD. The profile reactor is a versatile and accessible research tool for combined spatially resolved structure–activity profiling, enabling the use of multiple synchrotron-based characterization methods to promote a knowledge-based optimization of a wide range of catalytic systems in a time- and resource-efficient way.

## Abstract 2

This study introduces a catalytic profile reactor capable of simultaneously measuring spatially resolved temperature, concentration and X-ray diffraction profiles through a catalytic fixed bed under operation. The profile reactor is a versatile and accessible research tool, allowing the usage of multiple synchrotron-based characterization methods to understand and systematically optimize a wide range of catalytic systems.

## 1. Introduction

A major goal in modern heterogeneous catalysis research is to optimize catalysts and catalytic reactors based on an in-depth understanding of the processes involved. This optimization approach represents a time- and resource-efficient alternative to trial-and-error experiments often applied in conventional catalyst test systems (‘tail-pipe’ end analysis) (Hagmeyer *et al.*, 2004 ▸; Morgan *et al.*, 2016 ▸). A crucial step towards this goal is to unravel complex catalyst structure–activity relationships in order to tune the material properties and process conditions for the required chemical outcome. *Operando* measurements are a common tool to reveal such complex catalyst structure–activity relationships, probing catalysts in their active state under industrially relevant reaction conditions to provide species concentration, temperature, and spectroscopic and scattering information (Topsøe, 2003 ▸; Bañares, 2005 ▸; Frenken, 2017 ▸). However, experimental complexity is a considerable challenge. Catalytic processes occur on broad time- and length-scales, and thus a combination of multiple characterization methods is required to obtain complementary information at all relevant scales (Weckhuysen, 2009 ▸; O’Brien *et al.*, 2011 ▸; Grunwaldt *et al.*, 2013 ▸; Meirer & Weckhuysen, 2018 ▸;). Furthermore, precise control of reaction conditions in *operando* experiments is crucial, since catalysts are dynamic systems which are strongly influenced by temperature, pressure, flow velocity and concentration (Grasselli & Sleight, 1991 ▸; Bell, 2003 ▸; Newton, 2008 ▸; Schlögl, 2015 ▸; Zhou *et al.*, 2018 ▸). Spatial gradients in the aforementioned variables occur in almost all catalytic reactors under working conditions (Horn, 2020 ▸), although such gradients remain neglected in most studies. Thus, the development of suitable *operando* reactors coupled to spatially resolved measurement techniques has come strongly into focus, with the aim to provide simultaneous data on catalyst structure and reaction progress (Urakawa & Baiker, 2009 ▸; Grunwaldt *et al.*, 2009 ▸; Geske *et al.*, 2013 ▸; Dixon & Deutschmann, 2017 ▸; Wolf *et al.*, 2021 ▸).

The particular synergy between *operando* catalyst characterization and the versatile methods available at synchrotron light sources has resulted in many important methodological developments. One example is the standard quartz capillary micro-reactor (CMR) with plug-flow geometry. The CMR is compatible with diverse X-ray characterization tools and is almost universally applied to measure small powder samples (Grunwaldt *et al.*, 2006 ▸; Maurer *et al.*, 2020 ▸; Becher *et al.*, 2021*a* ▸; Alizadehfanaloo *et al.*, 2021 ▸). A more recent development is the aRCTIC setup, which provides a flexible sample environment for *operando* hard X-ray tomography of millimetre-scale samples using a rotatable CMR (Becher *et al.*, 2021*b* ▸). Although spatially resolved information of the catalyst structure can be rather easily obtained by moving the entire reactor system perpendicular to the X-ray beam, measurements of spatially resolved gas compositions require insertion of a gas sampling device [*e.g.* capillary sampling technique (Horn *et al.*, 2006 ▸)] in the catalyst bed, and thus larger reactor diameters (>4 mm). Hence, the small sample sizes of CMRs (typically <1.5 mm diameters) restrict concentration and temperature measurements to the reactor inlet and outlet, while there is no access to the dynamic and transient chemistry occurring within the catalyst bed. Larger reactor diameters limit the applicability of absorption techniques, whereas X-ray diffraction (XRD) or tomographic techniques using high energies can feasibly be applied on larger reactor diameters, as shown in several studies (O’Brien *et al.*, 2009 ▸; Wragg *et al.*, 2012 ▸; Vamvakeros *et al.*, 2018 ▸; Vamvakeros *et al.*, 2020 ▸; Matras *et al.*, 2021 ▸). However, these studies, as is the case for most spatially resolved synchrotron *operando* studies, measure only the catalyst in a spatially resolved manner. Simultaneous spatially resolved structure–activity measurements within the same reactor involve high experimental complexity, but have the potential to track catalyst structure and activity as a function of local chemical environments with high accuracy.

So far this was demonstrated by simultaneous spatially resolved concentration, temperature and X-ray absorption fine structure (XAFS) (Stewart *et al.*, 2018 ▸; Decarolis *et al.*, 2021 ▸; Wollak *et al.*, 2022 ▸) measurements through a catalytic fixed bed. In two of these studies, the spatially resolved capillary inlet reactor system for fixed beds and XAFS (SPACI-FB-XAFS) was used, in which larger sample sizes up to 4 mm [inner diameters (IDs)] and catalyst bed lengths of up to 12 mm were applied. One drawback, as with other contemporary reactor setups, involves the use of hot-air blowers as heating systems similar to those used in CMRs. Even though the applied hot-blower was equipped with a custom-designed nozzle (Goguet *et al.*, 2017 ▸), Newton *et al.* (2019 ▸) demonstrated that uniform heating by means of hot air is difficult to achieve. This is especially problematic with larger reactors and sample sizes, and can feasibly result in unrealistic or unrepresentative structural and chemical gradients due to non-uniform process conditions. One further drawback of most reactor systems is that a large number of individual parts need to be assembled and integrated at the beamline. This is relevant due to strict time constraints for setup assembly and usually limited space for accommodation of large setups at the beamline.

Limitations with prior setups therefore stimulated the development of the synchrotron Compact Profile Reactor (CPR) described in this study. The original CPR was developed for spatially resolved structure–activity profiling using Raman spectroscopy [one optical access (Wolf *et al.*, 2021 ▸)] under kinetically well defined, uniform and industrially relevant conditions, whereas the synchrotron CPR is optimized for X-ray absorption spectroscopy (XAS) [two optical accesses (Wollak *et al.*, 2022 ▸)]. The CPR has an integrated design, consisting of motors for sample movement, sample and trace heating, temperature measurements in the center of the catalyst bed and interior of the reactor, and a cooling system. Uniform sample heating of a 55 mm-long catalyst bed is achieved via direct contact between the reaction tube and a heating block. In earlier work (Wollak *et al.*, 2022 ▸), the power of spatial profiling in catalyst activity and *operando* XAS studies linked to the development of kinetic models was demonstrated. The kinetic model considers the Mo oxidation state to be a catalyst property, which could be validated with the experimentally observed oxidation states via Mo *K*-edge XAS. Overall, only very few studies of this kind are known in the literature, with previous applications limited to XAS as a possible characterization method. Although XAS allows the extraction of bulk metal atom oxidation states and coordination numbers, highly complementary methods such as XRD enable the deduction of crystalline phases in both a qualitative and a quantitative manner. The highly beneficial combination of XRD and XAS was shown to be an ideal tool to promote a holistical understanding of catalysts (Ressler *et al.*, 2000 ▸; Frenkel *et al.*, 2011 ▸; Gaur *et al.*, 2019 ▸, 2020 ▸; Müller *et al.*, 2020 ▸). Nowadays, XAS/XRD is increasingly gaining relevance through advanced beamlines, allowing simultaneous measurement of absorption and diffraction data for highly efficient characterization.

The goal of this work is to extend the combined methodological approach for simultaneous spatially resolved gas composition, temperature and diffraction profile measurements. *Operando* measurements were conducted using the synchrotron CPR for XRD experiments with a maximum opening angle of 2θ up to 32° for the scattered signal. The oxidative de­hydrogenation of ethane to ethyl­ene (ODH) over a 30 wt% MoO_3_ catalyst supported on γ-Al_2_O_3_ was chosen as a case study. This catalytic system shows distinct structural dynamics visible by changes in color as a function of reaction environment, and is a useful benchmark to demonstrate the power of the spatial profiling methodology. The measurement concept introduced offers a versatile and accessible approach for combined spatially resolved structure–activity profiling by means of synchrotron-based X-ray techniques to promote time- and resource-efficient optimization of catalytic reactors and catalysts.

## 2. Experimental

**Figure 1:**
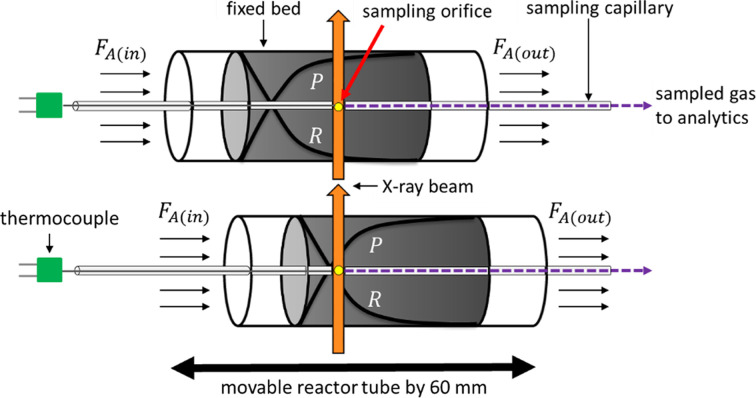
Working principle of the spatial profile measurement technique. *F* denotes the molar flow rate of a chemical species in the reaction mixture.

The CPR, developed by REACNOSTICS GmbH, was used to simultaneously measure spatially resolved gas composition, temperature and XRD through the catalytic fixed bed. Spatial gradients were obtained by the capillary sampling technique, schematically illustrated in Fig. 1 ▸. Here, a sampling capillary with a small sampling orifice runs through the center of a catalyst bed, placed in a reactor tube. Gas is continuously extracted from the reaction zone through the sampling orifice. A thermocouple sits inside the sampling capillary, tip-aligned with the sampling orifice. The capillary, and therefore the sampling orifice and thermocouple, are fixed in the space while the reactor tube is translated along the probed sample volume. In this way the entire catalyst bed can be moved along the measuring region comprising a sampling orifice, thermocouple tip and X-ray beam, allowing us to measure spatially resolved profiles. The X-ray beam was positioned next to the capillary to collect scattering information from the catalyst bed only and not from the sampling capillary. A detailed description of the capillary sampling technique is given in our previous work (Wollak *et al.*, 2022 ▸).

**Figure 2:**
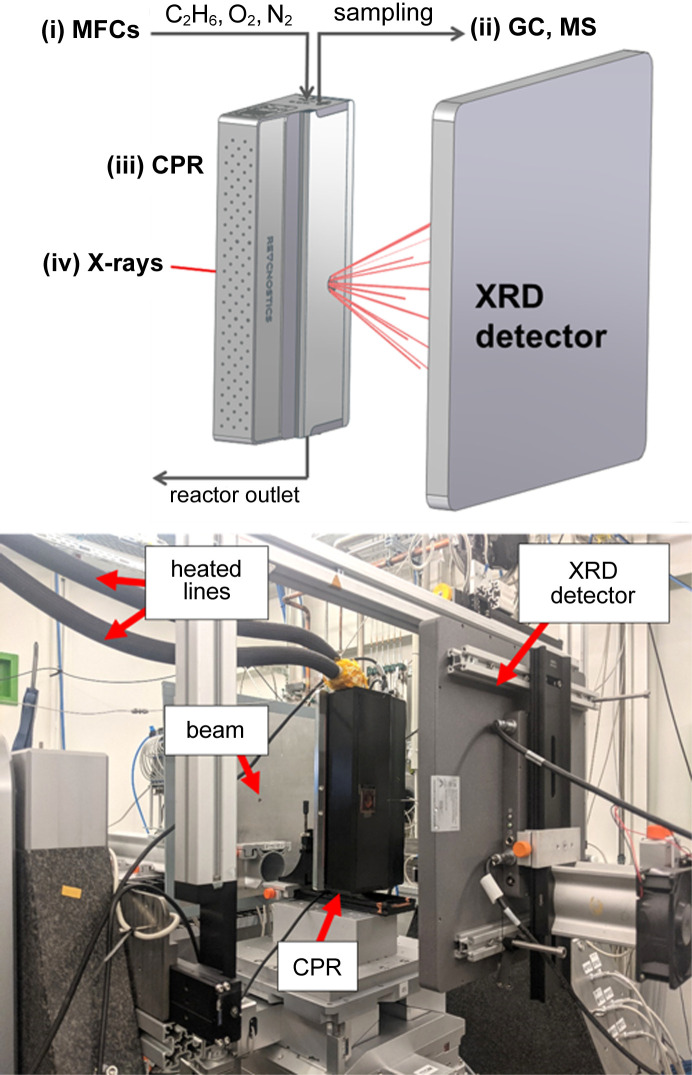
Overview of the experimental setup used for *operando* spatial profiling. (Top) Schematic representation: (i) gas dosing: set of MFCs; (ii) gas analytics: GC, MS; (iii) spatial profile reactor: CPR; (iv) XRD detector. (Bottom) Photograph of beamline P07 at PETRA III (DESY, Hamburg).

The experimental setup consists of four main parts: (i) a set of mass-flow controllers (MFCs), (ii) CPR, (iii) gas chromatograph (GC) or mass spectrometer (MS), and (iv) an XRD instrument (Fig. 2 ▸, top). A photograph of the installed setup at beamline P07 at PETRA III is given in Fig. 2 ▸ (bottom).

Precise dosing of the feed mixture was achieved by a set of MFCs (Bronkhorst GmbH). The gases were heated to the desired reaction temperature before reaching the beginning of the catalyst bed, which was assured by a preheating zone length of 4 cm within the CPR (iii). In order to reduce the invasiveness of the sampling methodology on hydro­dynamic conditions, only small sampling flow rates, adjusted by a micrometering (needle) valve (Valco Instruments Co. Inc. Vici AG International) to approximately 5% of the total flow rate, were continuously extracted and analyzed. Gas analysis was performed by a GC (Agilent 7890B) and an MS (Hiden HPR 20), including the measurement of condensable compounds such as water owing to the heating concept consisting of heated transfer lines (Hillesheim GmbH) and a heated reactor housing up to 200°C.

Spatial gradients inside the catalyst bed during the reaction were obtained by a modified CPR optimized for high-energy XRD. Here, optical access was achieved by cutting an opening (incident X-rays) and slit (exiting X-rays) in the heating block, which covers the sample tube, allowing measurements of scattered X-rays in transmission mode with a maximum solid angle 2θ up to 32°. The reaction tube (quartz tube Ilmasil PN9, QSIL GmbH) was vertically mounted inside the profile reactor to prevent bypass flows. A photograph of the inside of the CPR (Fig. S1 of the supporting information) as well as several drawings (Figs. S2–S4) are provided in the supporting information. The reaction temperature was measured in the center of the catalyst bed by a sheath-thermocouple type K using Inconel as sheath material [outer diameter (OD) 250 µm, TMH GmbH]. The latter was placed in a stainless steel sampling capillary (OD 700 µm, ID 520 µm, EHM Edelstahl GmbH) that has four side-sampling orifices, each laser drilled with a diameter of 75 µm, and arranged with an angular offset of 90° at the same axial position (Laser­Micronics GmbH). Thermocouple alignment within the stainless steel capillaries was achieved by employing the X-ray eye (Rischau, 2009 ▸) available for alignment at beamline P07. The maximum error in thermocouple position was 1.5 mm and thus all error bars in the temperature data are set to a fixed value of 1.5 mm in position.

Structural changes of the MoO_3_ catalyst were studied by high-energy XRD at the PETRA III storage ring at Deutsches Elektronen-Synchrotron (DESY) in Hamburg, Germany, at beamlines P21.1 and P07. High energies were required to minimize beam attenuation through the rather thick reactor tube of the CPR with an OD of 6 mm and an ID of 5–5.6 mm, as well as to minimize divergence for the exit beam to pass through the sample window in the reactor. The experimental setup (i)–(iv) can be fully automated due to the CPR control system (REACNOSTICS GmbH).

### 2.1. Catalyst activity tests

**Figure 3:**
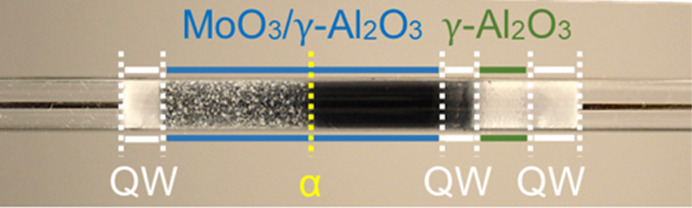
Reactor tube loaded with catalyst (MoO_3_/γ-Al_2_O_3_), support material (γ-Al_2_O_3_), sampling capillary as well as quartz wool (QW) to hold the packing in place. Alpha (yellow dotted line) marks a change in color of the catalyst, herein known as the color inflection point.

All profile measurements were performed using a 30 wt% MoO_3_ catalyst supported on γ-Al_2_O_3_ with a particle size of 300–400 µm. Catalyst preparation and characterization were described thoroughly in a previous publication (Wollak *et al.*, 2022 ▸). The catalyst was packed in a reaction tube made of fused silica with a 6 mm outer diameter, illustrated in Fig. 3 ▸.

The OD was constant while the ID varied depending on the wall thickness of the reactor tube used in the respective experiments (P07: 200 µm, ID 5.6 mm; P21.1: 500 µm, ID 5 mm). Catalyst bed lengths of 30 mm (P21.1) and 38 mm (P07) were applied. A gas composition of C_2_H_6_/O_2_/inert:10/10/80 and total flow rates of 12 ml min^−1^ (P21.1) and 15 ml min^−1^ (P07) with maximum gas hourly space velocities (GHSV) at the reactor outlet of 1248 and 978 h^−1^ were used. The reaction temperature was controlled to 515°C at the inlet of the catalyst bed. Prior to performing profile measurements, the catalyst was given 90 min to reach steady state, confirmed by constant conversion and selectivity data obtained by GC analysis at the reactor outlet stream during pre-tests.

### 2.2. Gas composition analysis

The analyzed reaction mixture was composed of C_2_H_6_, C_2_H_4_, CH_4_, H_2_O, O_2_, H_2_, CO_2_, CO and inert N_2_ or Ar. Gaseous species were analyzed by GC at P21.1 and by MS at P07. The GC used in this study was equipped with two columns (Plot Q, Molsieve) for compound separation and a thermal conductivity detector (TCD) connected in series with a flame ionization detector (FID) for species detection. Hydrogen could not be detected in the installed configuration, since it has a very similar thermal conductivity to He, which was used as a carrier gas. Quantitative GC gas analysis was performed using the internal standard method, referenced to N_2_. Calibration curves were obtained for all target species, except H_2_O and H_2_, through preparation of known gas mixtures by the same set of MFCs used in this study. Hydrogen was calculated from the hydrogen atom species balance of the reaction mixture and H_2_O was calibrated using a one-point calibration obtained from the oxygen atom species balance. At each position within the catalyst bed three GC runs were recorded. The resulting errors bars are not visible in the profiles, since the error bars are in the size range of the data points for the obtained standard deviations. The carbon balance was closed with average deviations of 2% during oxidation and 5% with non-oxidative conditions. Qualitative MS analysis was used to monitor the following mass to charge ratios (*m*/*z*): C_2_H_4_ (27), CO (28), C_2_H_6_ (30), O_2_ (32), Ar (40) and CO_2_ (44). Signals at 27 and 28 were corrected to remove contributions from other hydro­carbons or CO_2_. MS results are shown in signal ratios solely in the supporting information.

### 2.3. High-energy XRD measurements

*Operando* high-energy XRD measurements were carried out at beamlines P21.1 and P07 at PETRA III (DESY, Hamburg). The energies of the incident X-rays were chosen by Si(111) single-bounce monochromators with fixed angles corresponding to 101.6 keV, λ = 0.1220 Å (P21.1); and 103.6 keV, λ = 0.1199 Å (P07). The beam sizes were reduced and made square-shaped by slits to 0.8 mm × 0.8 mm (P21.1) and 0.5 mm × 0.5 mm (P07) to prevent hitting the stainless steel capillary during translation of the reactor bed. XRD patterns were recorded in transmission mode over a 2θ range from 1.3° to 11° using a 2D 410 mm × 410 mm Perkin Elmer XRD1621 detector with a pixel size of 200 µm × 200 µm (P21.1), and a 2D 432 mm × 432 mm Varex Imaging XRD 4343RF detector with a pixel size of 150 µm × 150 µm. At each sample position within the catalyst bed, first a dark image was recorded and afterwards 15 sample images were recorded with an exposure time of 60 s each, which were averaged for further analysis. Calibration of the sample-to-detector distances was performed using LaB_6_ (P21.1) and CeO_2_ (P07) as standards giving 1255 mm (P21.1) and 1170 mm (P07). Azimuthal integration was performed using the *pyFAI* package (Kieffer *et al.*, 2020 ▸). For qualitative phase analysis *HighScore Plus* software and the ICDD database were used (Degen *et al.*, 2014 ▸). An example structure refinement was performed for patterns recorded in the absence of gas phase oxygen using the *FullProf* software (FullProf, 2014 ▸; Rodríguez-Carvajal, 1993 ▸) based on the MoO_2_ oxide structure (ICSD code 23722).

## 3. Results and discussion

### 3.1. Spatially resolved concentration and temperature profiles

Results obtained from spatially resolved concentration and temperature profiles measured simultaneously with XRD on a 30 wt% MoO_3_/γ-Al_2_O_3_ catalyst during ethane ODH are the focus of the following section. Species concentration profiles analyzed by GC at beamline P21.1 and by MS at P07 are in good agreement, and comparable with a spatial profile study described in our previous work (Wollak *et al.*, 2022 ▸). However, reliable species quantification by MS is restricted to oxygen conversion levels up to 60%, because at higher oxygen conversion levels instabilities of the MS (*e.g.* signal response behavior) occur that result from a corresponding change in the oxidative properties of the reaction mixture. Thus, MS species profiles are shown in Fig. S6 of the supporting information, whereas the more accurate GC profiles are discussed in this section.

**Figure 4:**
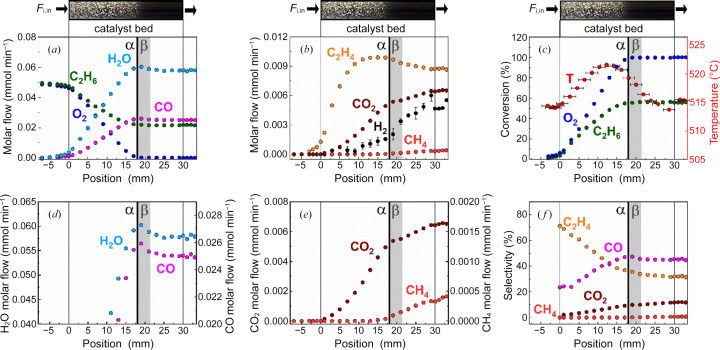
Image of the catalyst bed (top) with the inlet molar flow rate of each species (*F*_i,in_) and the catalyst activity profiles measured *operando* at beamline P21.1. Species concentration profiles of (*a*) C_2_H_6_, O_2_, H_2_O and CO; (*d*) H_2_O and CO; (*b*) C_2_H_4_, H_2_, CO_2_ and CH_4_; (*e*) CO_2_ and CH_4_; (*c*) conversion profiles of C_2_H_6_ and O_2_; (*f*) selectivity profiles of C_2_H_4_, CO, CH_4_ and CO_2_; and (*c*) temperature profile measured in the center of the catalyst bed. α (black line, 18 mm) marks the position of full gas phase oxygen conversion with different catalyst performance before and after. β (gray area) marks the catalyst bed range where the catalyst undergoes distinct phase transformations (see Section 3.2). Reaction conditions: C_2_H_6_/O_2_/N_2_:10/10/80, 515°C, 1 bar, OD 6 mm/ID 5.0 mm, 30 mm catalyst bed, 12 ml min^−1^, 30 wt% MoO_3_/γ-Al_2_O_3_.

Fig. 4 ▸ displays images of the catalyst bed (top) with the inlet flow rate (*F*_i,in_) from left to right, as well as an overview of the activity profiles obtained within one *operando* profile measurement (P21.1).

The profile measurement effectively allows us to discriminate between the different gaseous reactants and products and their concentrations at each internal position of the catalyst bed. Species profiles of C_2_H_6_, O_2_, CO and H_2_O [Fig. 4 ▸(*a*)]; and C_2_H_4_, H_2_, CO_2_ and CH_4_ [Fig. 4 ▸(*b*)] are shown as molar flow rates, and the corresponding conversion [Fig. 4 ▸(*c*)] and selectivity [Fig. 4 ▸(*f*)] profiles are shown as percentages. Enlarged views of H_2_O and CO [Fig. 4 ▸(*d*)], and CO_2_ and CH_4_ [Fig. 4 ▸(*e*)] profiles are also shown to emphasize characteristic profile shapes before and after the point of full gas phase oxygen conversion. The latter matches with the position of the inflection point (α, 18 mm) and is further referred to as α as well. The catalyst bed begins at position 0 and ends at position 30 mm, marked with black lines and illustrated by the corresponding photographs of the catalyst beds.

Notably, precise calibration of the reaction system is confirmed in Fig. 4 ▸(*a*) by almost equimolar inlet compositions (C_2_H_6_:O_2_ = 1:1). In the regions upstream (−7 to −5 mm) and downstream (31–34 mm) of the catalyst bed, only minor changes in educt consumption [Fig. 4 ▸(*a*)] or product formation [Fig. 4 ▸(*b*)] are visible (flat profiles), whereas steep gradients are evident along the catalyst bed (1–30 mm), confirming that ethane ODH only occurs in the presence of the catalyst at the chosen reaction conditions.

Close to position 0 mm (−4, −3, −2, −1), small changes are observable due to a mixed zone between quartz wool and catalyst, resulting from bed packing (likewise at ∼30 mm) as well as diffusional effects. The latter has stronger influence on profile shapes of lighter molecules such as H_2_, discussed in more detail later in this section.

Measurements performed behind the catalyst bed (positions >30 mm) are equivalent to the information obtained by conventional integral reactor analysis (reactor outlet measurements). However, gas concentration and temperature information within the reaction zone (0–30 mm) are not accessible using integral reactor analysis. In contrast, by employing the demonstrated capillary sampling technique, differential gas and temperature measurements through the catalyst bed (within the range of the black lines) could be acquired. This allows us to follow a broad conversion range of C_2_H_6_ (0–50%) and O_2_ (0–100%) within one profile measurement [Fig. 4 ▸(*c*)].

A combination of local sampling and reactor outlet measurements (differential and integral analysis) is particularly favorable during long profile runs to ensure an overall stable catalytic performance (*e.g.* catalyst deactivation) of less well known reaction systems. Stable catalytic performance as well as stable and reproducible operation of the profile reactor and the profile measurement technique used in this study were demonstrated in a long-term measurement campaign under various reaction conditions using a statistically well defined experimental design plan (Wollak *et al.*, 2022 ▸).

By evaluating the course of the entire species profiles within the reaction zone, a rapid identification of ongoing reactions and pathways can be obtained. As an example, the catalyst system used can be distinguished in two reaction regimes, before and after position 18 mm, illustrated with a black line and denoted α. The latter corresponds to the point of full gas phase oxygen conversion [Fig. 4 ▸(*c*)] and color inflection from gray to dark blue/black of the catalyst [picture of the catalyst bed, Fig. 4 ▸ (top)]. In the presence of gas phase oxygen (0–18 mm), ODH and oxidation reactions are predominant. O_2_ and C_2_H_6_ are readily consumed [Figs. 4 ▸(*a*) and 4 ▸(*c*)], while mainly CO and H_2_O [Fig. 4 ▸(*a*)], as well as small amounts of C_2_H_4_ and CO_2_ [Fig. 4 ▸(*b*)], are formed.

The product selectivity profile of ethyl­ene [Fig. 4 ▸(*f*)] decreases with an increase in ethane conversion [along the catalyst bed, Fig. 4 ▸(*c*)] while selectivity towards the undesired carbon oxides CO and CO_2_ increases. These profile shapes are as expected since the direct oxidation of ethane or further oxidation of ethyl­ene is likely to occur, both reducing ethyl­ene selectivity at higher ethane conversion levels.

By evaluating initial formation rates (initial profile slopes *m*_IPS_) of C_2_H_4_, CO and CO_2_ at the very beginning of the catalyst bed from −3 to 1 mm, information about primary (C_2_H_4_, CO *m*_IPS_ > 0) and secondary (CO_2_ *m*_IPS_ ≃ 0) products can be obtained. In this way, first reaction pathways can be proposed, but are restricted to a superficial understanding of the reaction network. To illustrate this point, it cannot be concluded whether either C_2_H_6_ or C_2_H_4_ is the main contributor towards the undesired CO_*x*_ production, or whether CO_2_ is produced in a consecutive reaction from CO. For this purpose, combined kinetic modeling using entire spatially resolved reactor data demonstrated its power in the fast development of kinetic models and the ability to reveal such reaction pathways. Using this approach and the same reaction system, we have shown in a previous study that CO originates mainly by a consecutive reaction step from C_2_H_4_ (Wollak *et al.*, 2022 ▸). The described kinetic model is developed for oxidation chemistry and therefore reactions occurring beyond α are not considered.

In addition to C_2_H_4_ and CO_2_, hydrogen occurs in minor quantities in the oxidation zone [Fig. 4 ▸(*b*)], *viz.* upstream of point α, where gas phase oxygen is still present. As discussed below, H_2_ is chemically produced downstream of α and diffuses rapidly against the flow direction. H_2_ is a light-weight molecule and, because the diffusion coefficient of a molecule scales with the square root of the molecular weight, hydrogen diffuses more than three times faster than all other components present in the reaction mixture. At the applied low total flow rate inside the catalyst bed, the reactor Péclet number of H_2_ is as low as about 10, taking the bed length as a characteristic dimension. In fixed bed reactors, a low reactor Péclet number indicates that axial dispersion cannot be neglected compared with convection, resulting in considerable back mixing. Even though the H_2_ concentration profile shown in Fig. 4 ▸(*b*) was calculated from the hydrogen atom species balance and sums up measurement errors from all other species, the effect that hydrogen diffuses upstream of point α was also confirmed experimentally, for example, in our previous study using a micro-GC (Wollak *et al.*, 2022 ▸) and also in the MS data displayed in the supporting information of the present study [Fig. S6(*e*)]. In agreement with the calculated profile in Fig. 4 ▸(*b*), the measured profiles show an increase in H_2_ flow rate way upstream of the point of complete O_2_ conversion α and a distinct increase in slope beyond that point. Close inspection of the photograph of the catalyst bed displayed on top of each flow rate panel in Fig. 3 ▸ (also shown in higher quality in Fig. S5) shows a faint darkening of the catalyst bed from the point where H_2_ is first detected in the catalyst bed until the color inflection point α where the catalyst turns dark violet, almost black. In this second reaction zone (18–30 mm, downstream of α) the overall reaction mechanism changes. In the absence of gas phase oxygen ODH and oxidation reactions stop. As seen in the formation of a new species [CH_4_; Figs. 4 ▸(*b*) and 4 ▸(*e*)], an increasing slope of the H_2_ profile [Fig. 4 ▸(*b*)], continued CO_2_ formation [Figs. 4 ▸(*b*) and 4 ▸(*e*)] and the occurrence of maxima in the profiles of C_2_H_4_ [Fig. 4 ▸(*b*)], H_2_O and CO [Fig. 4 ▸(*d*)], the chemistry in the oxygen-free zone of the catalyst bed is entirely different from the zone in which gas phase oxygen was present. C_2_H_4_, H_2_O and CO each pass through a maximum close to point α because these species are formed upstream of α and consumed downstream of α. The species profile shapes are the result of several overlapping reactions that, in combination, can explain the observed trends. The profiles provide evidence that a water–gas shift reaction (WGSR) [equation (1)] occurs,

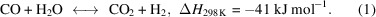

CO_2_ is the only carbon species that increases towards the end of the catalyst bed [Figs. 4 ▸(*b*) and 4 ▸(*e*)] with a smaller formation rate in comparison with the first reaction zone, further indicating a different formation reaction. In addition, H_2_ is formed [Fig. 4 ▸(*b*)] while CO and H_2_O decrease with a sharp onset at 18 mm [Fig. 4 ▸(*d*)].

Another reaction taking place in the oxygen-free zone of the catalyst bed is most likely ethyl­ene steam reforming, indicated by the decreasing C_2_H_4_ molar flow rate combined with profile shapes of H_2_O, H_2_ and CO,

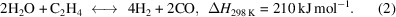

Finally, at position α methane production is observable, which suggests that CO or CO_2_ methanation [equations (3)–(4)] starts as soon as H_2_ is formed,

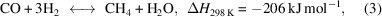




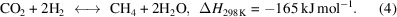

The spatially resolved carbon balance closure evaluated over the entire reaction zone (0–30 mm) demonstrates an upward trend from 0% to 6%, indicating the occurrence of additional minor side reactions, resulting in unidentified gas species or C deposits. The latter might originate from CO (Boudouard reaction) [equation (5)] or C_2_H_4_ [equation (6)],

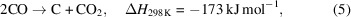




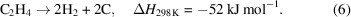

The profile measurement in this study was conducted at 515°C, which corresponds to the temperature measured in the center and upstream of the catalyst bed. The local temperature profile measured along the catalyst bed is shown in Fig. 4 ▸(*c*). The temperature rises slightly within the catalyst bed with a maximum value of 522°C around α, confirming that exothermic oxidation reactions are taking place.

These combined local temperature and gas phase analyses demonstrate their potential through high information content even within a single profile run. The observed changes in both the reaction mechanism and the color of the catalyst are inseparably linked to the structural changes in the catalyst. *Operando* XRD measurements were simultaneously performed during profile measurements to unravel the changes in the catalyst structure.

### 3.2. Spatially resolved *operando* high-energy XRD

**Figure 5:**
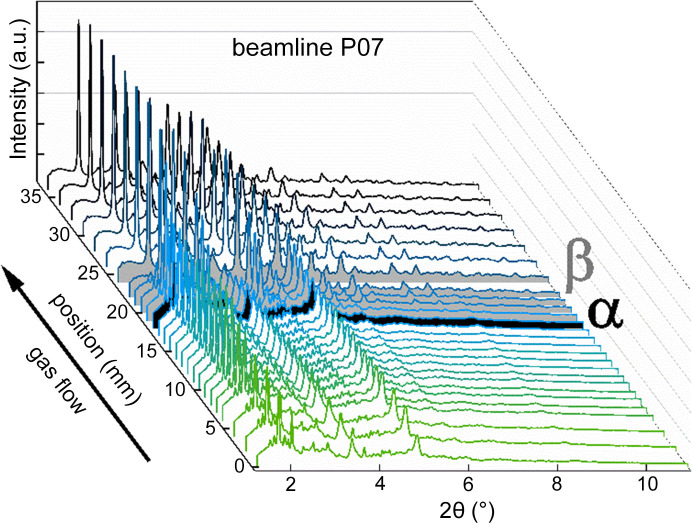
*Operando* XRD profile simultaneously measured with temperature and gas concentration profiles at beamline P07, PETRA III (Hamburg, Germany) during ethane ODH. α (black colored pattern, 18 mm) marks the position of full gas phase oxygen conversion with different catalyst performance before and after. β (gray colored patterns, 18–24 mm) marks the catalyst bed range where the catalyst undergoes distinct phase transformations. Reaction conditions: C_2_H_6_/O_2_/N_2_:10/10/80, 515°C, 1 bar, OD 6 mm/ID 5.6 mm, 38 mm catalyst bed, 15 ml min^−1^, 30 wt% MoO_3_/γ-Al_2_O_3_, beam size 0.5 mm × 0.5 mm (H × V), 103.4 keV (λ = 0.1199 Å).

The previous section showed that the catalyst was exposed to strongly varying local gas compositions with increasing reaction progress along the catalyst bed. This resulted in a change of the reaction mechanism and visual appearance of the catalyst at full gas phase oxygen conversion (α). However, these observations consider only one part of the chemical reaction system. Therefore, combined spatially resolved XRD was performed to analyze how the catalyst adapted in its crystalline phases along the catalytic bed, corresponding to the local chemical environment. Measurements were carried out at beamlines P07 and P21.1 (PETRA III, DESY) to develop and test the applicability of the new *operando* XRD spatial profiling methodology. XRD results from both beamlines compare well with each other in terms of the main features. Therefore, the following section will only focus on the XRD data obtained at P07. Results from P21.1 are shown in Figs. S7–S9. An overview of the XRD patterns obtained simultaneously measured with local catalyst activities are presented in Fig. 5 ▸.

**Figure 6:**
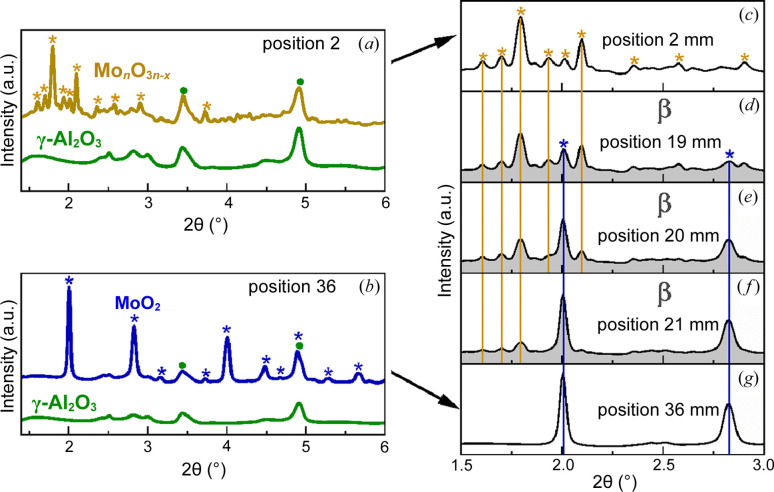
XRD patterns measured *operando* at beamline P07, PETRA III (Hamburg, Germany). (*a*, *c*) Diffractogram at the beginning of the catalyst bed (yellow, 2 mm, before β) in the presence of gas phase oxygen; (*b*, *g*) diffractogram at the end of the catalyst bed (blue, 36 mm, beyond β) in the absence of gas phase oxygen. (*a*, *b*) Each pattern contains signal contributions from fused silica and alumina. A corresponding pattern is shown in green. (*d*)–(*f*) XRD patterns measured at the catalyst bed position β (18–24 mm). Reaction conditions: C_2_H_6_/O_2_/N_2_:10/10/80, 515°C, 1 bar, OD 6 mm/ID 5.6 mm, 38 mm catalyst bed, 15 ml min^−1^, 30 wt% MoO_3_/γ-Al_2_O_3_, beam size 0.5 mm × 0.5 mm (H × V), 103.413 keV (λ = 0.1199 Å).

Here, 27 diffractograms were recorded at the same positions as the sampling points of the species concentration profiles, which form the corresponding XRD profile through the 38 mm-long catalyst bed. The catalyst bed can be separated into three zones (0–18 mm; β: 18–24 mm; 24–38 mm) based on structural similarities. XRD patterns before α (0–18 mm) and after β (24–38 mm) are very similar, showing qualitatively the same reflections, whereby the first bed zone shows changes in the signal-to-background ratio and peak intensities, and the third zone shows varying peak intensities, discussed later in this section. In the second bed zone, denoted β, XRD reveals a pronounced phase transformation, which starts at the point of full gas phase oxygen conversion (α, 18 mm) obtained by the species concentration profiles [Figs. S6(*a*) and S6(*d*)]. An overview of the phase transformation is illustrated in Fig. 6 ▸.

The diffractograms measured before and after position β are represented by the patterns at positions 2 mm and 36 mm, respectively, which are shown in the 2θ range 1.3–6° on the left [Figs. 6 ▸(*a*) and 6 ▸(*b*)], and with a smaller angular region of 1.5–3° on the right [Figs. 6 ▸(*c*) and 6 ▸(*g*)]. Further, patterns acquired inside the transition zone β at 19 mm [Fig. 6 ▸(*d*)], 20 mm [Fig. 6 ▸(*e*)] and 21 mm [Fig. 6 ▸(*f*)] are shown in the stack plot (right). Each diffractogram has signal contributions from the reaction tube (fused silica) and support material (γ-Al_2_O_3_). A corresponding pattern of the reactor tube filled with pure support material is shown in Figs. 6 ▸(*a*) and 6 ▸(*b*), illustrated in green. Fused silica shows a characteristic broad shoulder in the 2θ range 1.4–2°, while the strongest reflections from γ-Al_2_O_3_ occur at 3.5° {004} and 4.9° {044} [ICDD code 98–003–0267]. The as-prepared calcined sample exhibits a yellow color at 515°C in O_2_/N_2_:20/80. On reaction the catalyst quickly changed color from yellow to gray at the beginning of the catalyst bed. The corresponding sample diffraction patterns measured at 2 mm [Fig. 6 ▸(*a*)] cannot be assigned to either the previous MoO_3_ phase nor another obvious phase mixture. Molybdenum oxides are known to form numerous intermediate oxides, such as Mo_5_O_14_ [ICDD code 98–007–2639], Mo_18_O_52_ [ICDD code 98–002–7510], Mo_17_O_47_ [ICDD code 0.98–002–8333], Mo_9_O_26_ [ICDD code 98–003–8014], Mo_8_O_23_ [ICDD code 98–020–2203] and Mo_4_O_11_ [ICDD code 98–002–4033]. The main reflections originating from the aforementioned phases occur in the low 2θ range 1.6–2.2° or show very low intensities. The ability of molybdenum oxides to rapidly exchange gaseous oxygen leads to the formation of various suboxides, small crystallite sizes and an oxygen-defective structure, the extent of which is thermodynamically determined by temperature and the local oxygen partial pressure. Oxygen defects cause peak-broadening as well as small crystallites, which decrease resolution and might lead to overlapped or hidden reflections, making phase identification challenging. Though the XRD patterns show a stable phase mixture over the measurement time at one bed position, the patterns between the sampling positions in the first reaction zone show varying signal-to-background ratio and peak intensities. Here, the strongly decreasing oxygen concentration is the result of a varying phase composition along the catalyst bed, consisting of a mixture of molybdenum (sub)oxides with a changing crystallite size and number, including the formation of XRD amorphous phases.

The XRD patterns obtained beyond β [36 mm, Fig. 6 ▸(*b*)] show crystalline MoO_2_ with a monoclinic crystal structure (ICDD code 98–015–2316) as the only crystalline phase related to molybdenum oxide. The onset of Mo_*n*_O_3*n*–*x*_ reduction to MoO_2_ is observed at position 19 mm [Fig. 6 ▸(*d*)] through the appearance of MoO_2_ reflections and at the same time decreasing Mo_*n*_O_3*n*–*x*_ signals. At 24 mm no reflections corresponding to non-stoichiometric oxides are detected.

**Figure 7:**
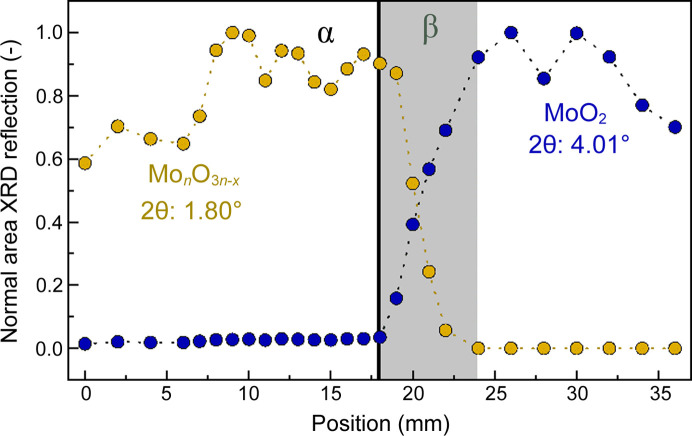
Area evaluation of reflections at 1.80° (corresponding to Mo_*n*_O_3*n*–*x*_) as well as at 4.01° (corresponding to MoO_2_). The areas were normalized to the maximum area obtained at the respective reflections. α (black line, 18 mm) marks the position of full gas phase oxygen conversion with different catalyst performance before and after. β (gray area, 18–24 mm) marks the catalyst bed range where the catalyst undergoes distinct phase transformations. Reaction conditions: C_2_H_6_/O_2_/N_2_:10/10/80, 515°C, 1 bar, OD 6 mm/ID 5.6 mm, 38 mm catalyst bed, 15 ml min^−1^, 30 wt% MoO_3_/γ-Al_2_O_3_, beam size 0.5 × 0.5 (H × V), 103.413 keV (λ = 0.1199 Å).

The course of the phase transformation is further addressed by the evaluation of peak areas at 2θ of 1.80° and 4.01°, showing no overlap and strong reflections corresponding to Mo_*n*_O_3*n*–*x*_ and MoO_2_, respectively (Fig. 7 ▸).

There is a noticeable sharp decrease in Mo_*n*_O_3*n*–*x*_ and increase in MoO_2_ from 18 mm to 24 mm, which complements previous observations of catalyst reduction. The oscillation in the signals upstream and downstream of this region are observed similarly in the XRD profile measured at P21.1, without showing the same oscillatory trends, indicating a random variation. For both measurements of 15 images were recorded at each position, showing no significant deviations. Hence, XRD measurements were reproducible and showed a negligible statistical error. A possible explanation for the observed variations along the bed could be related to insufficient particle statistics in the XRD measurements, compared with optimal powder XRD analysis. With respect to the setup, the sample cannot be rotated. In addition, a compromise had to be found to fulfill the requirements of reaction engineering and XRD analysis, leading to the usage of rather large catalyst particles (300–400 µm). Therefore, effects resulting from crystal orientation (*e.g.* directional crystal growth at high temperatures) might be observed in the XRD profiles. Also minor effects like variations in the local Mo-loading and in the bed density might contribute to the observed deviations. Lastly, it might also be that the oscillations originate from actual changes in the phases, since the catalyst is exposed to strongly varying gas compositions along the catalyst bed. For example, in the first bed zone a growth in crystal numbers of a respective Mo_*n*_O_3*n*–*x*_ phase could result in changing XRD patterns, while at the end of the bed, beyond position 30 mm, the decreasing trend of the MoO_2_ phase could indicate a further reduction of MoO_2_.

**Figure 8:**
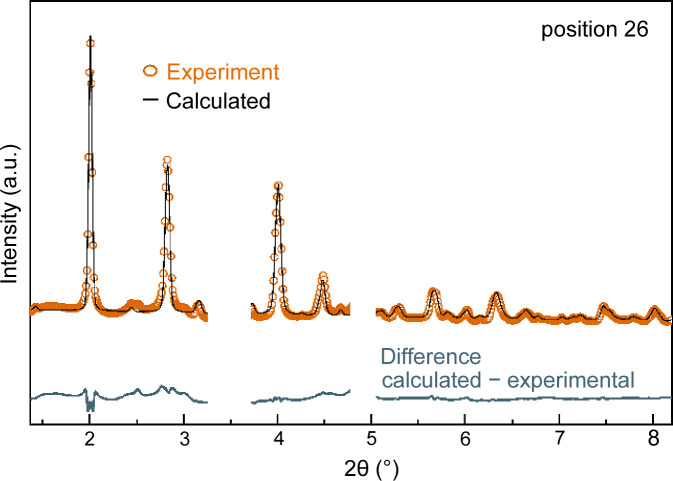
Experimental XRD pattern measured *operando* at position 26 mm and results of the Rietveld refinement (experimental data in orange, calculated in black, difference calculated–experimental in gray). The two areas around 3.5° and 4.9° have been masked out of the refinement because of the relatively large contribution from γ-Al_2_O_3_. Reaction conditions: C_2_H_6_/O_2_/N_2_:10/10/80, 515°C, 1 bar, OD 6 mm/ID 5.6 mm, 38 mm catalyst bed, 15 ml min^−1^, 30 wt% MoO_3_/γ-Al_2_O_3_, beam size 0.5 × 0.5 (H × V), 103.413 keV (λ = 0.1199 Å).

To illustrate the quality of the data obtained, a Rietveld refinement was performed for the XRD pattern at position 26 mm using the monoclinic structure of MoO_2_ (ICSD code 23722). A good match is achieved between experimental and calculated patterns (Fig. 8 ▸), demonstrating high data quality and enabling a thorough structure analysis, which is planned for future works.

In summary, *operando* XRD reveals a distinct phase transformation from a mixture of various Mo_*n*_O_3*n*–*x*_ phases towards a highly crystalline monoclinic MoO_2_ at full gas phase oxygen conversion. Catalyst reduction downstream of this position, as well as identified phases, are in line with color and catalytic performance observations, obtained in the previous section. Molybdenum (sub-)oxides are known to be active towards de­hydrogenation and oxidation reactions (Kube *et al.*, 2017 ▸; Ressler, 2002 ▸; Heracleous *et al.*, 2004 ▸), while it is reported that MoO_2_ has metallic character (Katrib *et al.*, 1996 ▸), which makes it a suitable catalyst for WGSRs and methanation reactions (SAITO, 1980 ▸, 1981 ▸).

Combined spatially resolved *operando* XRD adds an additional dimension of data that allows us to correlate structural and mechanistic information of the catalyst at work based on the position within the reactor. Using the synchrotron CPR, the data measured were acquired under precisely controlled reaction conditions, essential for accurate deduction of catalyst structure–activity relations in *operando* studies. The precise control of operation conditions over a wide range allows, in combination with the applicability of several (multiple) characterization methods, systematic studies of various reaction systems in the future.

## 4. Conclusions

The introduced *operando* measurement concept enables simultaneous acquisition of spatially resolved activity and structural information of heterogeneous catalytic systems at work. Here, for the first time, temperature, gas composition and high-energy XRD profile measurements were performed simultaneously through a catalytic fixed bed. The methodology was validated using ethane ODH to ethyl­ene over a 30 wt% MoO_3_/γ-Al_2_O_3_ catalyst as a test reaction.

By means of the synchrotron CPR, *operando* studies under kinetically well defined, uniform and industrially relevant reaction conditions were realized. The high data quality enabled quantitative gas and crystalline phase analyses of the identified phases which enabled us to follow the chemical evolution within the catalyst bed. The integrated and fully automated setup enables time saving during assembly, compatibility with a range of beamlines, as well as straightforward sample changeover and operation, which is particularly beneficial in catalysis studies at synchrotron radiation facilities. In addition, the technique is widely applicable for a broad range of reaction systems, including those with challenging requirements such as high pressure and temperature, as well as for a variety of spectroscopic and scattering catalyst characterization methods, such as XRD, XAS, Raman spectroscopy, total scattering, SAXS and related method types.

In future works, simultaneous studies with multiple characterization techniques – in particular XRD/XAS – using the measurement concept introduced, are encouraged. XRD demonstrates its power to track long-range ordered structures, while XAS probes short-range ordered structures. In combination with local gas concentration analysis a more complete picture of the processes inside catalytic reactors can be obtained. Such data are required to develop and validate precise kinetic models for systematic, knowledge-based process optimization.

This study expands the portfolio of spatially resolved *operando* synchrotron-based catalyst characterization methods to XRD. The strength of correlative structure–activity profiling is demonstrated by providing numerous insights within one profile measurement, illustrating a promising approach to promote efficient optimization of heterogeneously catalyzed reactions in the future.

## Supplementary Material

Supporting Figures S1 to S9. DOI: 10.1107/S1600577523001613/ye5030sup1.pdf
