# Supplementary material for: Catalytic reactor for operando spatially resolved structure–activity profiling using high-energy X-ray diffraction
Source: J Synchrotron Radiat. 2023 Apr 12;30(Pt 3):571–81. doi: 10.1107/S1600577523001613 (PMC10161877; doi:10.1107/S1600577523001613)
Supplement: Supplementary file 1 [file s-30-00571-sup1.pdf]

JOURNAL OF
SYNCHROTRON
RADIATION

**Volume 30 (2023)**

**Supporting information for article:**

# Catalytic Reactor for *Operando* Spatially Resolved Structure-Activity Profiling using High-Energy X-Ray Diffraction

**Birte Wollak, Diego Espinoza, Ann-Christin Dippel, Marina Sturm, Filip Vrljic, Olof Gutowski, Ida G. Nielsen, Thomas L. Sheppard, Oliver Korup and Raimund Horn**

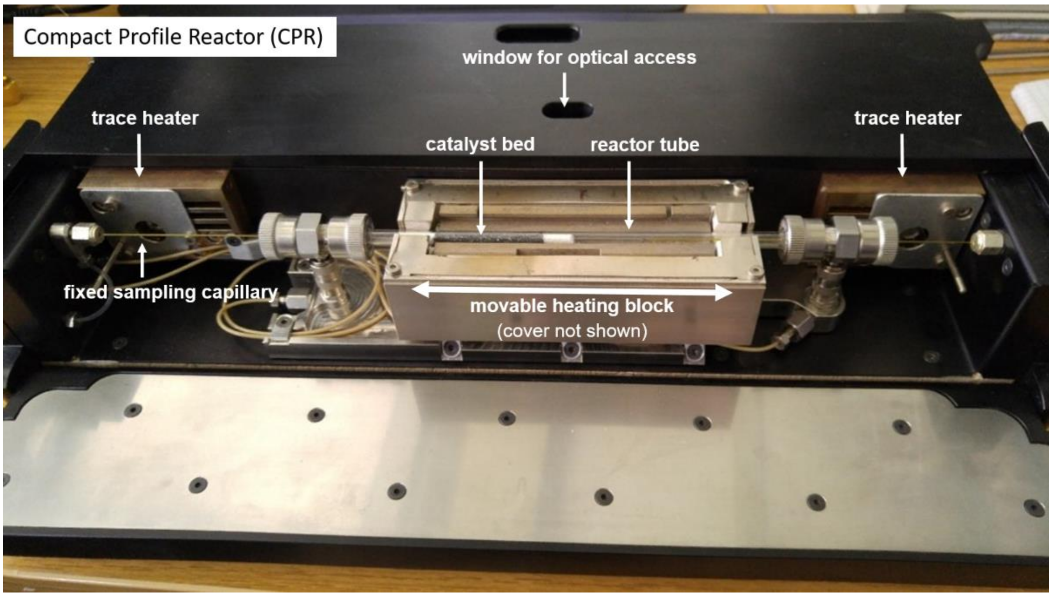


**Figure S1** A photograph of the inside of the CPR.

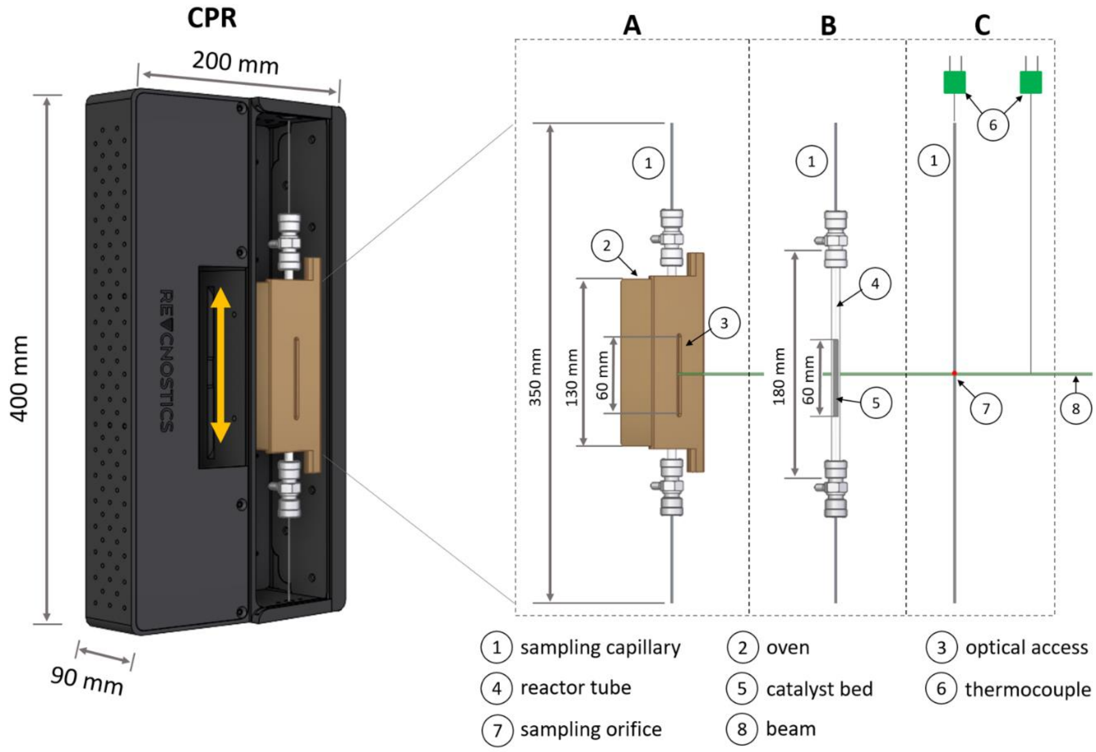


**Figure S2** Illustration of the CPR (left) and the components inside (A – C). A: An enlarged view of the heating block which covers the reactor tube and contains a slit-shaped optical access window. B: The reactor tube filled with the catalyst bed and the sampling capillary in the center. C: The sampling capillary with the thermocouple located inside. The green line represents the incident laser or X-ray signal, aligned with the sampling orifice, the thermocouple tip and the opening of the reactor block.

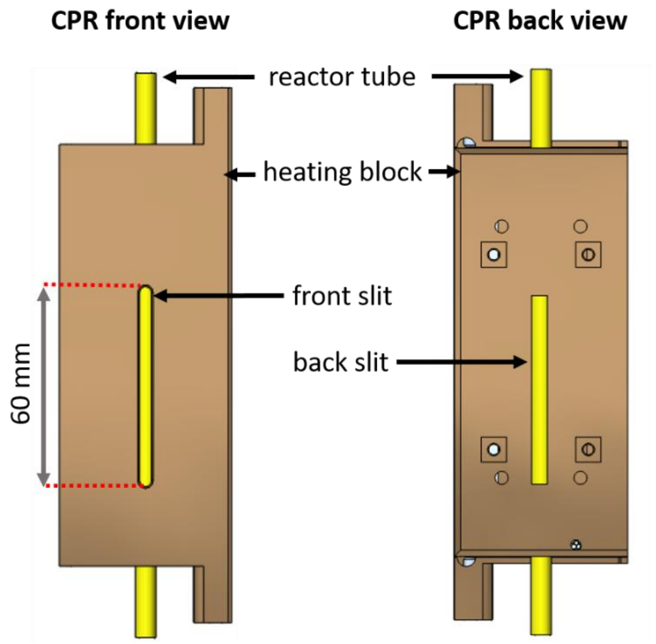


**Figure S3** Schematic of the sample heating block showing two openings. One on the front (left) and one on the back (right) to allow X-rays passing through the reactor tube.

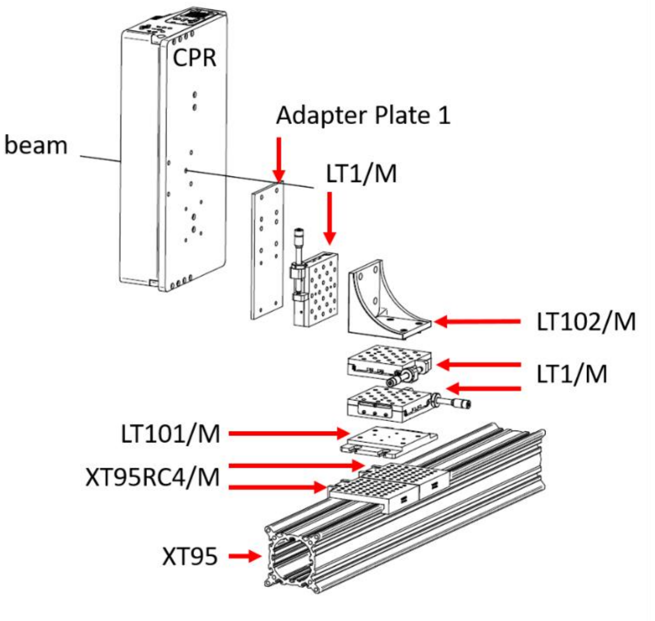


**Figure S4** Adapter system used to mount and align the reactor at several beamlines. The rail systems varies depending on the beamline infrastructure and specifications. Except of the Adapter Plate 1, all parts were purchased from Thorlabs GmbH. The Thorlabs parts comprise three single-axis translation stages that each provided a travel distance of 50 mm on the respective axis with high precision. In this way, a more flexible reactor positioning in x-y-z directions was given at different beamlines.

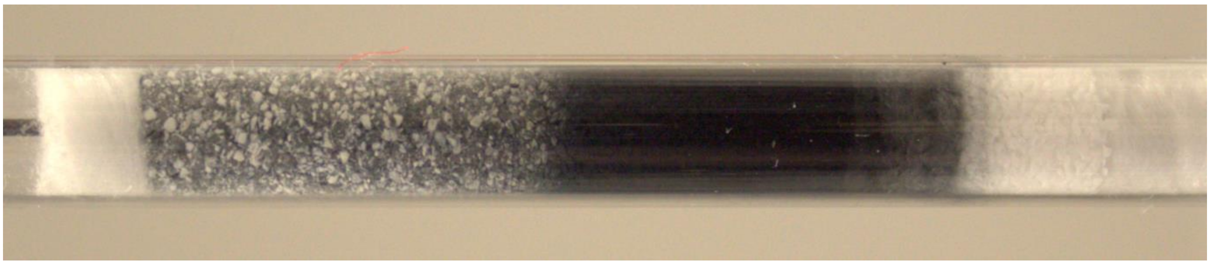

**Figure S5** A photograph of the catalyst bed after the reaction.

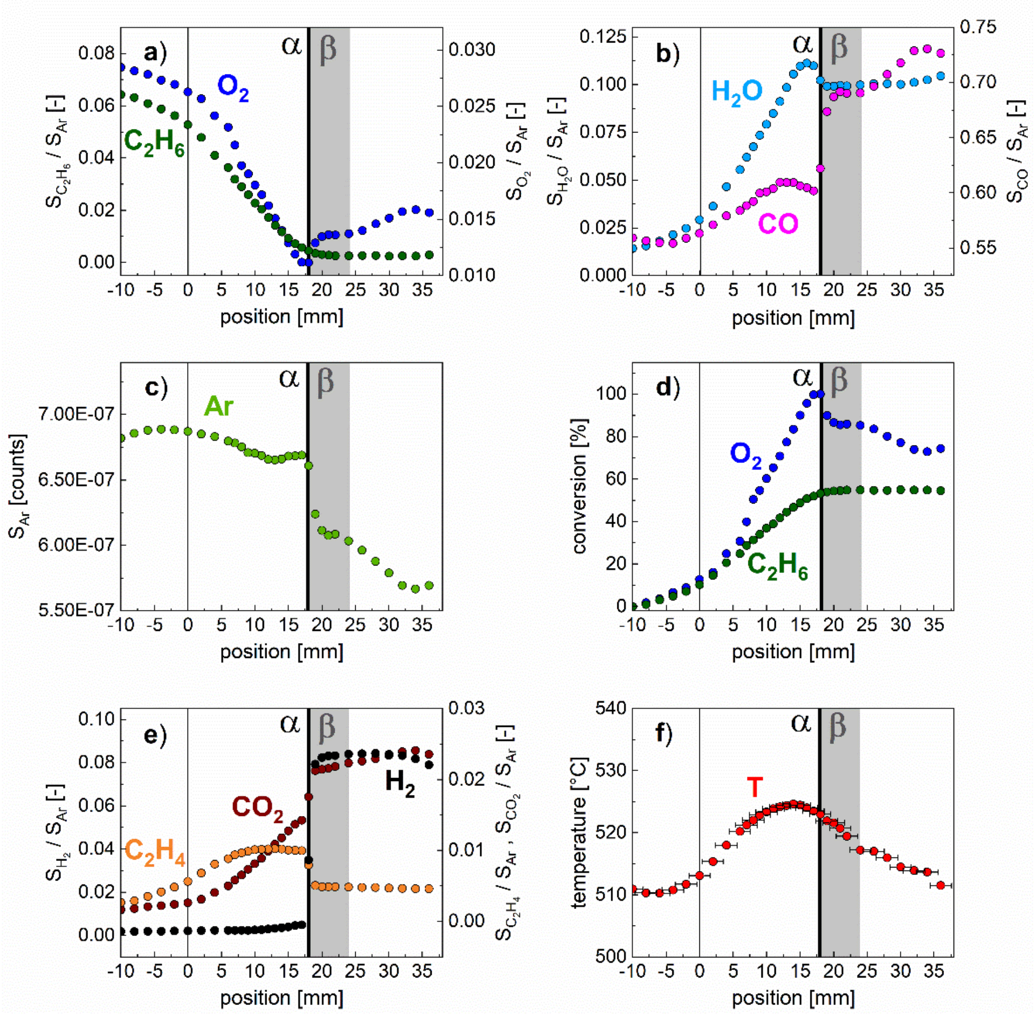


**Figure S6** Catalyst activity profiles measured operando at beamline P07 using a MS (a – f). Signal ratios of $C_2H_6$, $O_2$ (a), $H_2O$, CO (b), $C_2H_4$, $H_2$, $CO_2$ (c), inert Ar (d); conversion profiles of $C_2H_6$ and $O_2$ (e); and the temperature profile measured in the center of the catalyst bed (f). α (black line, 18 mm) marks the position of full gas phase oxygen conversion with different catalyst performance before and after. β (gray area, 17.5 – 21.5 mm) marks the catalyst bed range where the catalyst undergoes distinct phase transformations. Reaction conditions: $C_2H_6/O_2/N_2$:10/10/80, 515 °C, 1 bar, OD 6 mm/ID 5.6 mm, 38 mm catalyst bed, 15 ml/min, 30 wt% $MoO_3/\gamma\text{-}Al_2O_3$

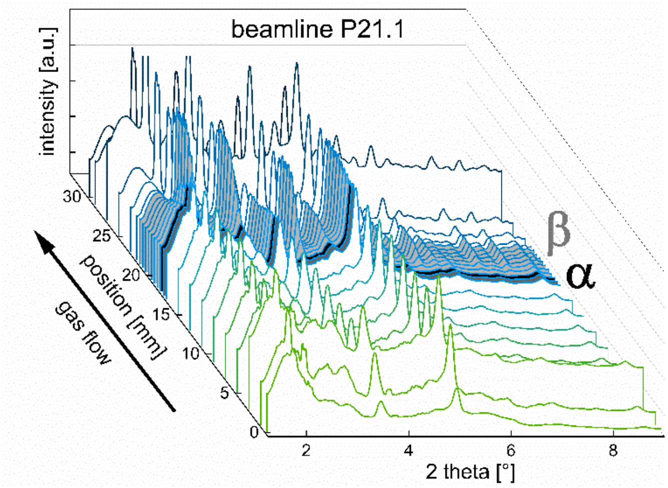


**Figure S7** Operando XRD profile simultaneously measured with temperature and gas concentration profiles at beamline P21.1, PETRA III (Hamburg, Germany) during ethane ODH. α (black colored pattern, 18 mm) marks the position of full gas phase oxygen conversion with different catalyst performance before and after. β (grey colored patterns, 17.5 – 21.5 mm) marks the catalyst bed range where the catalyst undergoes distinct phase transformations. Reaction conditions: $C_2H_6/O_2/N_2$:10/10/80, 515 °C, 1 bar, OD 6 mm/ID 5.0 mm, 30 mm catalyst bed, 12 ml/min, 30 wt% $MoO_3/\gamma\text{-}Al_2O_3$, beam size 0.5 × 0.5 $mm^2$ (h × v), 101.6 keV (λ = 0.1220 Å).

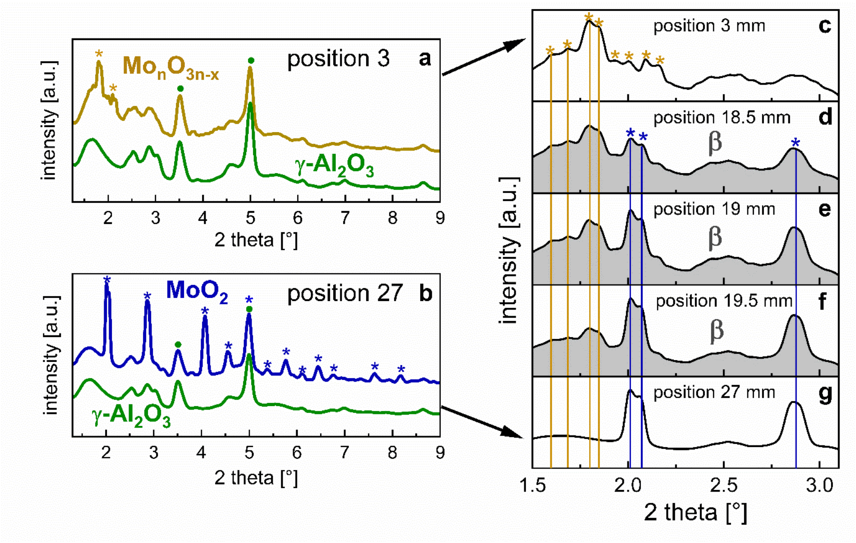


**Figure S8** XRD patterns measured operando at beamline P21.1, PETRA III (Hamburg, Germany). a, c) Diffractogram at the beginning of the catalyst bed (yellow, 3 mm, before β) in the presence of gas phase oxygen; b, g) Diffractogram at the end of the catalyst bed (blue, 27 mm, beyond β) in the absence of gas phase oxygen. a, b) Each pattern contains signal contributions from fused silica and alumina. A corresponding pattern is shown in green. d – f): XRD patterns measured at the catalyst bed position β (17.5 – 21.5 mm). Reaction conditions: $C_2H_6/O_2/N_2$:10/10/80, 515 °C, 1 bar, OD 6 mm/ID 5.0 mm, 30 mm catalyst bed, 12 ml/min, 30 wt% $MoO_3/\gamma\text{-}Al_2O_3$, beam size 0.5 × 0.5 $mm^2$ (h × v), 101.6 keV (λ = 0.1220 Å).

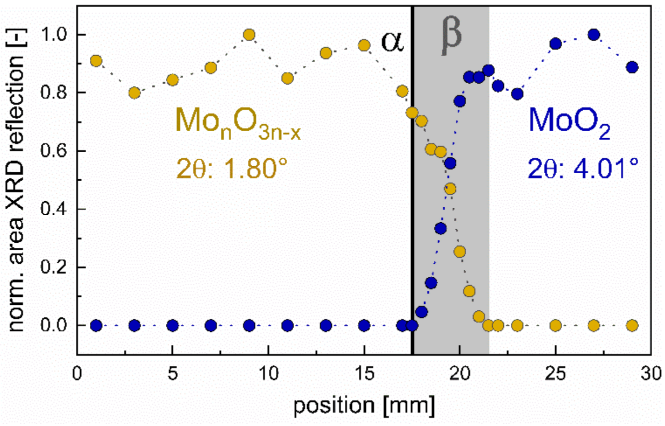


**Figure S9** Area evaluation of reflections at 1.80° (corresponding to $Mo_nO_{3n-x}$), as well as at 4.01° (corresponding to $MoO_2$) from P21.1 data. The areas were normalized to the maximum area obtained at the respective reflections. α (black line, 18 mm) marks the position of full gas phase oxygen conversion with different catalyst performance before and after. β (grey area, 17.5 – 21.5 mm) marks the catalyst bed range where the catalyst undergoes distinct phase transformations. Reaction conditions: $C_2H_6/O_2/N_2$:10/10/80, 515 °C, 1 bar, OD 6 mm/ID 5.0 mm, 30 mm catalyst bed, 12 ml/min, 30 wt% $MoO_3/\gamma\text{-}Al_2O_3$, beam size 0.5 × 0.5 $mm^2$ (h × v), 101.6 keV (λ = 0.1220 Å).
